# Reduced graphene oxide as a stable and high-capacity cathode material for Na-ion batteries

**DOI:** 10.1038/srep40910

**Published:** 2017-01-18

**Authors:** Ghulam Ali, Asad Mehmood, Heung Yong Ha, Jaehoon Kim, Kyung Yoon Chung

**Affiliations:** 1 Center for Energy Convergence Research, Korea Institute of Science and Technology, Hwarang–ro 14–gil 5, Seongbuk–gu, Seoul 02792, Republic of Korea; 2 Korea University of Science and Technology, 217 Gajeong–ro Yuseong–gu, Daejeon 34113, Republic of Korea; 3 School of Mechanical Engineering & SKKU Advanced Institute of Nano Technology, Sungkyunkwan University, 2066, Seobu-ro, Jangan-Gu, Gyeong Gi-do 16419, Republic of Korea

## Abstract

We report the feasibility of using reduced graphene oxide (RGO) as a cost-effective and high performance cathode material for sodium-ion batteries (SIBs). Graphene oxide is synthesized by a modified Hummers’ method and reduced using a solid-state microwave irradiation method. The RGO electrode delivers an exceptionally stable discharge capacity of 240 mAh g^−1^ with a stable long cycling up to 1000 cycles. A discharge capacity of 134 mAh g^−1^ is obtained at a high current density of 600 mA g^−1^, and the electrode recovers a capacity of 230 mAh g^−1^ when the current density is reset to 15 mA g^−1^ after deep cycling, thus demonstrating the excellent stability of the electrode with sodium de/intercalation. The successful use of the RGO electrode demonstrated in this study is expected to facilitate the emergence of low-cost and sustainable carbon-based materials for SIB cathode applications.

The development of advanced energy storage systems has become an important research area because of their vast usage in applications ranging from portable electronic devices to grid-level energy storage. Intermittent energy sources such as geothermal, solar, and wind require large-scale energy storage systems^1^. Lithium-ion batteries (LIBs) are dominant amongst the energy storage technologies for small- to medium-scale electronic devices. The use of electrical energy storage is expanding to large-scale applications, such as transportation and stationary storage systems. However, LIBs are not suitable for large-scale applications because of their high production cost and limited lithium resources. Sodium-ion batteries (SIBs) have emerged as a potential candidate for large-scale energy storage systems (ESS) because of their advantages of a low production cost and evenly distributed global sodium reserves compared to lithium^2^,^3^,^4^,^5^. For the successful application of SIBs, the electrodes should deliver high round-trip efficiency, a long cycle life and flexible power.

In recent years, several cathode materials such as layered oxides (NaMO_2_ (M = 3d transition metals) and their solid solutions)^6^,^7^,^8^,^9^,^10^, sulfates (e.g., NaFeSO_4_F, Na_2_Fe_2_(SO_4_)_3_, Na_2_Fe(SO_4_)_2_.2H_2_O)^11^,^12^,^13^, phosphates (e.g., NaFePO_4_, Na_3_V_2_(PO_4_)_3_, Na_2_FePO_4_F, Na_2_FeP_2_O_7_)^14^,^15^,^16^,^17^, fluorides (e.g., FeF_3_, NaFeF_3_, FeF_3_**·**0.5H_2_O)^18^,^19^,^20^, and Prussian blue (e.g., Na_2_MFe(CN)_6_·H_2_O and KMFe(CN)_6_ where M = transition metal, Na_2_Mn[Mn(CN)_6_])^21^,^22^,^23^, have been reported for SIBs. However, most of these materials either have a low sodium storage capacity (<200 mAh g^−1^) or undergo rapid capacity degradation over cycling. The composition of most of the cathodes used in SIBs include transition metals, which are not environmentally benign and are often costly. Furthermore, the difficult synthesis procedure, low electronic conductivity and complex structural arrangements during sodium de/intercalation process make layered transition metal oxides unsuitable for use in high-performance SIBs at their present stage of development^24^,^25^.

In that regard, inexpensive, environmentally friendly and highly conductive carbonaceous materials are of great interest as electrode materials for electrochemical energy storage devices such as SIBs, LIBs and supercapacitors^26^,^27^,^28^. Among them, graphene, due to its ultrathin two-dimensional structure, has unique properties such as high electrical conductivity, large surface area, and high chemical and mechanical stabilities, and has been used widely in various applications such as electrodes for energy storage systems, field effect transistors, sensors, and catalyst support^29^,^30^,^31^,^32^. Graphene has been extensively investigated for its use in electrochemical energy storage devices, such as serving an anode material for LIBs, due to its large capacity compared to the commonly used graphite electrodes. Graphene-based electrodes show higher capacities because they trap lithium ions by different mechanisms than graphite, such as double layer capacitance, adsorption and defect trapping^33^,^34^,^35^,^36^. Most of the work on graphene has mainly focused on its application as an anode material for LIBs. The approach to functionalizing the graphene oxide (GO) with the alkali-metal-ions have exhibited reasonable sodium storage capacity as anodes for SIBs with long cycle life of upto 600 cycles at a high current density of 1 A g^−1^ 37. GO as a composite anode with Sb showed a high sodium storage capacity of 220 mAh g^−1^ at an ultra high current density of 12 A g^−1^ 38. A more stable barrier layer is expected to form upon sodium insertion in reduced graphene oxide (RGO), which prevents further oxidation and leads to a better stability compared to lithium insertion. Some studies have shown that sodium ion can de/intercalate into RGO. Almost all such investigations on RGO in SIBs have considered anodes only^39^,^40^. In the present study, for the first time, we conduct a comprehensive performance evaluation of RGO as an SIB cathode to demonstrate the potential of this inexpensive and abundantly available class of materials in SIB technology. The RGO is prepared by mixing the as-prepared graphene oxide and graphene nanosheet powder at a ratio of 9:1 and reducing it by microwave irradiation. The RGO electrode is cycled in an upper voltage range of 1.2–4.5 V, which makes it suitable for use as a cathode. The RGO cathode shows a high sodium storage capacity of 240 mAh g^−1^ at a current density of 30 mA g^−1^ over 1000 cycles, which indicates the excellent stability of this material.

## Results

**Figure 1:**
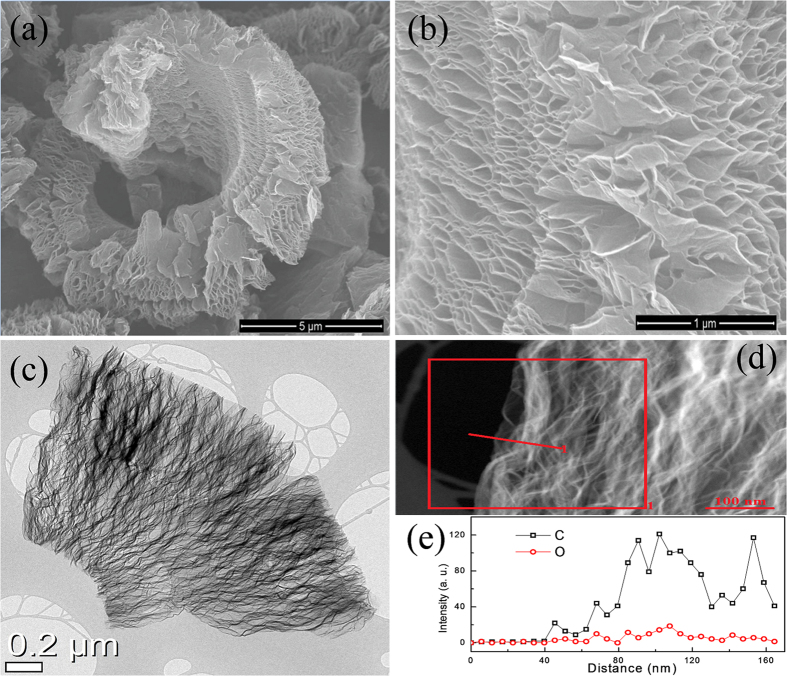
Morphological and structural characterization of RGO. SEM images at (**a**) low and (**b**) high magnification. (**c**) TEM images of the RGO stack. (**d**) EDS line-profile showing the functional groups on RGO and (**e**) relative signals of C and O from the EDS line-profile image.

**Figure 2:**
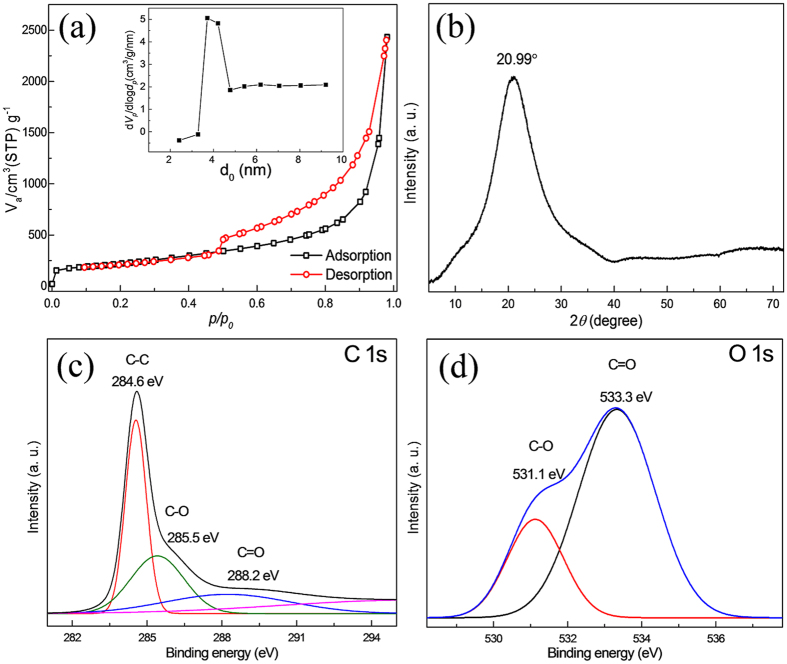
Surface and structural characterizations. (**a**) Nitrogen adsorption/desorption isotherm; the inset shows the pore size distribution as measured by the BJH model. (**b**) XRD pattern plotted with respect to Cu-Kα (1.54 Å). XPS spectra of (**c**) C 1s and (**d**) O 1s.

The morphological and physical characteristics of the reduced graphene oxide prepared by modified Hummer’s method^41^ are shown in Fig. 1. From Fig. 1a and b, a highly wrinkled stack of ultra-thin graphene oxide nanosheets with a porous morphology can be observed (Fig. 1a). The nanosheets prepared by a solid-state microwave irradiation method are connected to a vermiculite morphology that is analogous to the thermal exfoliation of graphene oxide^42^. A high-magnification SEM image (Fig. 1b) shows large pores between the nanosheets that are formed by gas evolution. These interconnected pores are extended from one side of stack to the other along the length of the nanosheets. Figure 1c shows a TEM image of RGO stack that is expanded at the outer edge and squeezed at the inner side, thus revealing a segment of doughnut-like morphology. Figure 1d and e show the EDS line-profile, which demonstrates the existence of functional groups on the RGO. The relative contents of C and O were detected and plotted against distance (Fig. 1e). The average atomic ratios of C and O were measured to be 91.2 and 8.8 at.%, respectively. The contents show that the RGO has an extensively functionalized surface and could act more effectively for charge storage. The surface area and porosity of RGO were examined using the nitrogen physiosorption method, and the adsorption-desorption curves given in Fig. 2a indicate a type-IV isotherm that is a representative of mesoporous structures. The specific surface area and total pore volume of the RGO nanosheets, as determined by the Brunauer–Emmett–Teller (BET) method, are 789 m^2^ g^−1^ and 3.86 cm^3^ g^−1^, respectively. Such a high surface area and large porosity indicate that RGO nanosheets hold a well-exposed surface for the electrochemical reactions. The Barrett-Joyner-Halenda (BJH) pore-size distribution curve (inset of Fig. 2a) indicates that the diameter of the majority of the pores is between 3.3 and 4.8 nm. In general, the pore structure and BET surface area of graphene-based materials depend on the open structure between the 2D nanosheets. The RGO nanosheets show a prominent pore diameter peak centered at 3.72 nm, which is expected to be beneficial for enhancing the electrochemical reactions because it can facilitate the penetration of electrolytes into the interior of the material, resulting in a larger ionic transportation. X-ray powder diffraction was used to characterize the RGO powder as shown in Fig. 2b. The XRD pattern shows a broad peak at a 2*θ* value of ~20.99°, which indicates a disordered two-dimensional structure with stacked layers of RGO nanosheets^40^. The corresponding interlayer distance of RGO nanosheets is 4.3 Å (calculated from d-spacing), which is sufficiently large for the insertion/extraction of sodium ions. The increased *d*-spacing interlayer distance and high surface area of RGO indicate that interlayer distance is expanded after the intercalation of sulfuric acid and the reduction of graphene oxide. X-ray photoemission spectroscopy (XPS) was used to observe the composition and chemical structure of RGO. Figure 2c shows a high-resolution C 1s XPS spectrum of the RGO nanosheets. The sharp peak at 284.6 eV corresponds to C-C bond, whereas other peaks at high energies of 285.5 and 288.2 eV can be attributed to different C-O and C = O bonds, respectively. The oxygen-containing functional groups in RGO were further confirmed by the O 1s spectrum (Fig. 2d). The deconvoluted spectrum shows two main peaks of C-O and C = O at 531.1 and 533.3 eV, respectively. The carbon and oxygen contents were measured to be 91.39 and 8.61 at.%, which correspond to a high carbon to oxygen ratio (C/O = 10.6) and are in agreement with the EDS results quantities.

**Figure 3:**
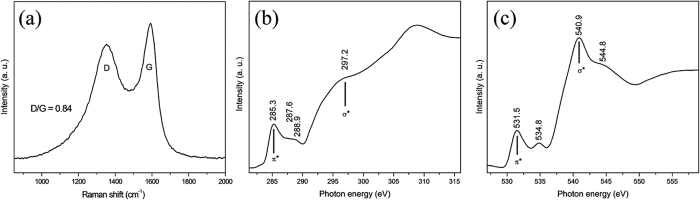
Structural characterizations. (**a**) Raman spectra (D and G bands) of the RGO sample. NEXAFS spectra of (**b**) the C K-edge and (**c**) the O K-edge for the RGO powder.

Raman spectroscopy is a non-destructive and direct technique for characterizing the quality and structure of graphene^32^,^43^. The Raman spectrum of RGO was collected at an excitation wavelength of 514 nm, and it shows two major peaks at 1352 and 1592 cm^−1^ (Fig. 3a), corresponding to the typical D-band and G-band, respectively. The position of both bands depends strongly on the grain size, defects, edges, disorder and microstructure of the carbonaceous materials^44^. The G-band reflects the hexagonal structure associated with the E_2g_ vibration mode of sp^2^ hybridized carbon atoms, and the D-band is related to the disordered A_1g_ breathing mode of the six-fold aromatic ring near the basal edge and structural defects of the sp^2^ domains^32^,^45^. The high intensity of the G-band shows that RGO is not completely exfoliated and contains stacked graphitic layers, as also shown in the SEM and XRD results. The D-/G-band ratio reflects the degree of disorder in RGO, and it was measured to be 0.84, which indicates that the ordered hexagonal structure is several nanometers in size^45^. The defective nature of carbon structure could also enhance the adsorption, which is favorable for the sodium storage mechanism^46^.

The electronic structure of RGO was determined using synchrotron-based, high-resolution NEXAFS spectroscopy, which is an effective method for studying the unoccupied states of a specific material. Figure 3b shows the C K-edge NEXAFS spectrum of RGO, where the electronic transition occurs from the core level to unoccupied states into π* C = C symmetry around the K and M points of graphene oxide layers and the corresponding peak occurs at 285.3 eV. The higher energy peak at 297.2 eV can be attributed to the excitation of C 1s electron into σ* C-C symmetry^47^. The relative peak intensity (I_π*_/I_σ*_) of both resonances after reduction provides a measure of the electronic structure restoration, and it was calculated to be 0.47. The low intensity of π* is related to the oxidation step of RGO, which indicates the partial recovery of π conjugation in the RGO^48^. The peaks at 287.6 and 288.9 eV can be attributed to the transitions from the core level to the π* C-O and to π* C = O unoccupied states, respectively, which are localized in the functional groups at the edges and the basal planes^49^. Figure 3c shows the general trends in the O K-edge NEXAFS spectrum of RGO. The peak at 531.5 eV can be attributed to the transition from O 1s core level to unoccupied states with π* C = O resonance; this belongs to the carboxyl groups attached at the edges and may have belonged to the carbonyl groups bonded to an aromatic ring^47^. The peaks at 534.8, 540.9 and 544.8 eV can be attributed to the π* C-O resonance from epoxides, σ* C-O resonance and σ* C = O resonance related to carbonyl groups, respectively, and the peaks beyond these reflect the total oxygen contents in the sample^47^,^50^. Overall, the NEXAFS spectroscopy results provide information on the partial recovery of the π-conjugated structure of the RGO with the removal of the oxygen functional groups.

**Figure 4:**
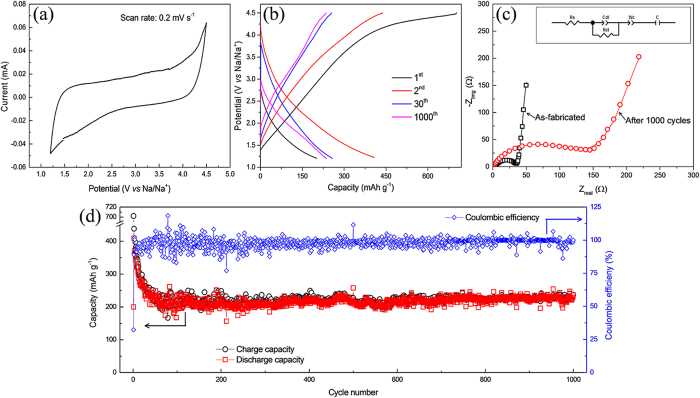
Evaluation of electrochemical performance of the RGO cathode. (**a**) CV scan at a scan rate of 0.2 mV s^−1^ in a voltage range of 1.2–4.5 V. (**b**) Charge-discharge profiles at a current density of 30 mA g^−1^ in the 1^st^, 2^nd^, 30^th^, and 1000^th^ cycles. (**c**) Electrochemical impedance spectroscopy analysis of as-fabricated and after 1000^th^ cycles of RGO cathode and the inset shows the circuit diagram, used to calculate the charge-transfer resistance. (**d**) Cycle stability in a voltage range of 1.2–4.5 V at a current density of 30 mA g^−1^.

The sodium-ion storage behavior of the RGO cathode was first evaluated using cyclic voltammetry (CV) at a scan rate of 0.2 mV s^−1^ in a voltage range of 1.2–4.5 V (Fig. 4a). It should be noted that CV is measured after five galvanostatic cycles to stabilize the formation of the solid electrolyte interface (SEI) film. The CV curve of the RGO cathode shows a broad and rectangular-shaped profile during the reduction/oxidation process, which are indicative of typical electric double-layer capacitive (EDLC) behavior^51^. The shape of the CV curve indicates that the reaction mechanism in the RGO cathode is confined to the surface reactions but is not diffusion-limited^52^. Galvanostatic measurements were performed at an initial current density of 30 mA g^−1^, and the charge-discharge profile of the RGO cathode is shown in Fig. 4b. The electrode exhibits a discharge capacity of 201 mAh g^−1^ during the 1^st^ sodiation process. This value of capacity shows a high sodium insertion, which can be attributed to the sodium adsorption on the RGO nanosheets and is much larger compared to the reported literature where the RGO electrode showed a discharge capacity of 78 mAh g^−1^ 53. The open structure of RGO could hamper the excessive aggregation of carbon nanosheets and extend the interface for the electrochemical reactions, ultimately leading to a large discharge storage capacity. During the recharge process, the electrode exhibits a large capacity of 702 mAh g^−1^, corresponding to a coulombic efficiency of 29%. The low coulombic efficiency during the first cycle is presumably attributed to the irreversible losses that are associated with the oxidative decomposition of electrolyte occurring on the electrode surface at higher potentials. Furthermore, a large gap is observed between charge and discharge curves, indicating high polarization which results in low energy density of the cell. The dis/charge profile of the RGO cathode shows sloping voltage profiles with significant voltage hysteresis, which demonstrates that sodium storage is mainly due to Na adsorption on RGO layers as already observed by CV. Furthermore, the potential profile changes gradually without any plateau during charge-discharge process, indicating that the electrochemical reaction at RGO cathode is based on fast surface reactions and this phenomenon resembles the electrochemical capacitors. Thus sodium insertion/extraction processes in RGO cathode are analogous to those of sodium-ion capacitors. The 2^nd^ cycle shows a high sodium insertion/extraction level during the discharge/charge process, and the obtained capacities during discharge and charge process are 408 and 438 mAh g^−1^, respectively, which correspond to a high coulombic efficiency of 93%. The higher sodium insertion contents can be attributed to the storage on both sides of RGO layers. However, both the charge and discharge capacities reduce with cycling and the capacity stabilize around the 30^th^ cycle, where the electrode shows a stable reversible capacity of 257 mAh g^−1^ (Fig. 4d). This is presumably due to the structural adjustments with successive Na insertion/extraction. The charge transfer resistance (R_ct_) was measured using electrochemical impedance spectroscopy (EIS) of as-fabricated and after 1000^th^ cycle of RGO cathode (Fig. 4c). The diameter of the semicircle in the Nyquist plot represents the R_ct_, followed by an inclined line describing Warburg impedance (W_c_). EIS spectra were fitted using equivalent-circuit as shown in the inset of the Fig. 4c and R_ct_ values were calculated to be 33 and 149 Ω for as-fabricated and cycled cells, respectively. This demonstrates that sodium ions face relatively higher polarizations with cycling. Considering the adsorption mechanism, it is expected that the RGO electrode could show a long cycle performance and good rate capability. The electrode exhibits stable capacity after the 30^th^ cycle and reveals an outstanding cycle performance for 1000 cycles, with a high coulombic efficiency of >90% (Fig. 4d). A reversible capacity of 236 mAh g^−1^ is obtained in the 1000^th^ cycle, thus demonstrating an excellent capacity retention of 92%. To the best of our knowledge, this is the best electrochemical performance of a RGO electrode with exceptional cyclic stability reported to date.

**Figure 5:**
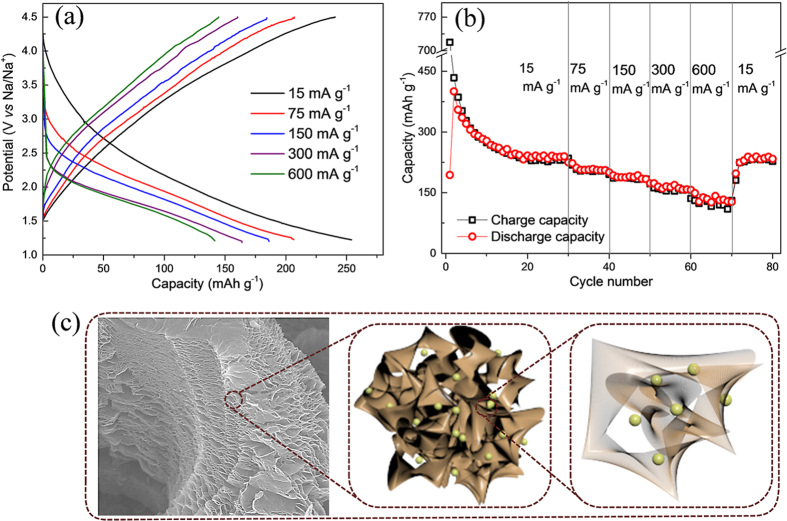
Galvanostatic rate performance and charge storage mechanism. (**a**) Charge-discharge profiles at variant current densities and (**b**) the corresponding rate capabilities of RGO cathode at current densities of 15, 75, 150, 300 and 600 mA g^−1^. (**c**) Schematic representation of sodium de/insertion into the RGO nanosheets, where sodium atoms are represented in green.

The rate capabilities of the RGO electrode were measured at current densities of 15, 75, 150, 300 and 600 mA g^−1^, and their resulting charge-discharge profiles are provided in Fig. 5a. A nearly sloping charge-discharge profile is observed at all current densities, which suggests a similar insertion mechanism. Average discharge capacities of 265, 208, 187, 163 and 134 mAh g^−1^ are obtained at current densities of 15, 75, 150, 300 and 600 mA g^−1^, respectively. These capacity values demonstrate the superior rate performance of the RGO electrode. After a deep cycling at 600 mA g^−1^, when the current densities are reset to 15 mA g^−1^, the electrode recovered a high discharge capacity of 230 mAh g^−1^ (Fig. 5b), which shows the promising structural stability of the material at high currents. Figure 5c presents a schematic depiction of the sodium insertion/extraction pathways through the large pores of the RGO nanosheets. The presence of oxygen contents (C = O functional groups) on the RGO surface could be responsible and act as a redox center for sodium storage at high potential. The high rate capability is the result of fast surface reactions on the RGO, which enable rapid sodium storage through open channels. An expanded interlayer distance and the larger sheet size of RGO together provide better junction contacts, which allow fast sodium insertion/extraction.

## Discussion

In summary, the as-synthesized RGO showed a mesoporous wrinkled structure with large pores of nanometer size and a high BET surface area of 789 m^2^ g^−1^. The TEM EDS line-profile coupled with XPS confirmed the existence of oxygen functional groups in RGO. The NEXAFS analysis provided useful information about the chemical bonding between carbon and oxygen atoms and the partial recovery of the π conjugated structure of RGO with the removal of oxygen groups. The honey-comb type multi-layer structured RGO showed an excellent electrochemical performance as a cathode, which can be attributed to the adsorption of sodium ions on the well-exposed surface of nanosheets. The RGO cathode exhibited a high discharge capacity of >235 mAh g^−1^ over 1000 cycles and maintained a high coulombic efficiency of >90%, thus demonstrating the good reversibility of the material. More importantly, the RGO electrode also showed a high rate capability, and it delivered a discharge capacity of 134 mAh g^−1^ at a high current density of 600 mA g^−1^, thus indicating the good stability of the electrode. Hence, we have shown the successful use of a carbonaceous material, which is expected to open new avenues for the development of low-cost and sustainable SIBs. These type of sodium deficient cathodes can be applied in practical use by either pre-sodiation or using sacrificial materials.

## Methods

Graphene nanosheets incorporating RGO were synthesized by a modified Hummers’ method. Graphite powder, potassium permanganate (99%), hydrogen peroxide (>30%) and sulfuric acid (>95%) were purchased from Sigma Aldrich. First, a mixture of 25 ml of concentrated sulfuric acid and 1 g of graphite powder was stirred, and 3.5 g of KMnO4 was poured slowly into the above solution at 0 °C. The mixture was then stirred at 35 °C for 2 h, followed by the addition of 40 ml of deionized water. After 1 h of stirring, hydrogen peroxide was continuously added to the mixture until no gas evolution was observed. The graphene oxide was collected using centrifugation and dried at 70 °C for 12 h. The as-prepared graphene oxide was reduced using the solid-state microwave irradiation method. Briefly, graphene oxide powder (90 wt.%) was mixed with graphene nanosheet powder (10 wt.%), which later acted as an effective microwave susceptor to produce high-quality RGO. The mixture was then transferred to a quartz tube in a glove box in an Ar atmosphere and was subsequently reduced by microwave irradiation using a microwave oven (Mars5, CEM). The microwave treatment was carried out at 1600 W in pulsed irradiation mode.

### Characterizations

The morphology of as prepared RGO was evaluated by field-emission scanning electron microscopy (FE–SEM, NOVA NanoSEM200, FEI). The Brunauer–Emmett–Teller (BET) method was used to measure the specific area of the RGO by nitrogen adsorption (77 K) using a BEL instrument (BEL, Japan). XRD was measured with Mo–Kα radiations (wavelength of 0.7107 Å) using a diffractometer (R–AXIS IV++, Rigaku) at the Korea Institute of Science and Technology (KIST). RGO powder was filled in a quartz capillary tube for measurement and the data were recorded on image plate. The data were converted to a 1D XRD pattern using the GSAS-II program^54^ and for analysis, the wavelength was converted to Cu–Kα radiation (wavelength of 1.54 Å). Raman spectrum was recorded using a Thermo electron Corporation (Nicolet Almega XR dispersive Raman) instrument. X-ray photoelectron spectroscopy (XPS) was conducted by PHI 5000 Versa Probe, Japan Analytical. NEXAFS spectra of C and O K-edges were taken at 10D KIST bending magnet beamline at Pohang Light Source (PLS-II). The sample was mounted on pure indium (In) foil and all the measurements were performed at room temperature. The spectra were collected in a total electron yield (TEY) mode under a base pressure of 3 × 10^−10^ Torr and 0.01 eV resolution. Each edge spectrum was measured at least five times and averaged to obtain high quality data.

### Electrochemical measurements

The cathodes were prepared by mixing the RGO, carbon black and polyvinylidene fluoride binder (PVDF) at a weight ratio of 6:2:2, and a proper amount of N–methyl–2–pyrolidinone (NMP) was added to the mixture. The slurry was cast onto an Al foil and roll–pressed after drying at 80 °C. Electrodes with a thickness of ~30 μm and active mass loading of ~0.6 mg cm^−2^ were punched and vacuum dried prior to fabricating the cells. Coin cells (CR 2032) were fabricated in an argon filled glove box (Mbraun Unilab, Germany) for the electrochemical measurements of the RGO electrodes. 1–M NaClO_4_ in a 1:1:1 (volume ratio) EC: PC:diethyl carbonate (DEC) solvent was used as an electrolyte, and sodium foil was used as a counter electrode. Cyclic voltammetry and electrochemical impedance spectroscopy were carried out using a Biologic potentiostat/galvanostat Model VMP3 (BioLab, Inc.), and the galvanostatic performances were tested on a battery cycler (Maccor 4000).

## Additional Information

**How to cite this article**: Ali, G. *et al*. Reduced graphene oxide as a stable and high-capacity cathode material for Na-ion batteries. *Sci. Rep.* **7**, 40910; doi: 10.1038/srep40910 (2017).

**Publisher's note:** Springer Nature remains neutral with regard to jurisdictional claims in published maps and institutional affiliations.
